# CHILD ABUSE AND ADULT MENTAL HEALTH: DOES GENDER MATTER?

**DOI:** 10.1093/geroni/igz038.3401

**Published:** 2019-11-08

**Authors:** Patricia M Morton, Blakelee Kemp, Frass Ahmed

**Affiliations:** 1 Wayne State University, Detroit, Michigan, United States; 2 Syracuse University, Syracuse, New York, United States; 3 University of Michigan, Ann Arbor, Michigan, United States

## Abstract

Numerous studies have demonstrated that child abuse is associated with poor adult mental health, but few have investigated the extent to which the frequency of different types of abuse increase mental health conditions, especially at the nexus of gender. The present study examines whether parental abuse frequency and abuse perpetrator have distinct effects for men and women on three mental health outcomes—depressive symptoms, generalized anxiety, and global self-reported mental health. Data came from three waves of the National Survey of Midlife Development in the United States (MIDUS), comprising a baseline sample of 3,032 adults aged 25-74. Estimating a series of mixed effects models revealed that maternal abuse and frequent abuse during childhood were associated with poorer adult mental health during our 20-year observation period, net of childhood and adult risk factors. Specifically, maternal emotional abuse raised the risk of depression, anxiety, and lower self-rated mental health, and was more strongly associated with depression and anxiety for women than men. Compared to adults who did not experience parental abuse during childhood, adults who experienced frequent emotional and physical abuse by either parent were more likely to experience depression and anxiety and report lower ratings of mental health in adulthood. Frequent child abuse was more strongly associated with anxiety for women than men. These results demonstrate that gender differences in adult mental health have early-life antecedents. Future research investigating the long-term mental health consequences of child abuse should consider the type and magnitude of abuse as well as the perpetrator.

